# Decreased telomere length in a subgroup of young individuals with bipolar disorders: replication in the FACE-BD cohort and association with the shelterin component POT1

**DOI:** 10.1192/j.eurpsy.2024.469

**Published:** 2024-08-27

**Authors:** L. Spano, C. Marie-Claire, O. Godin, M. Leboyer, B. Aouizerate, A. Lefrere, R. Belzeaux, P. Courtet, E. Olie, C. Dubertret, R. Schwan, V. Aubin, P. Roux, M. Polosan, L. Samalin, E. Haffen, F. Bellivier, B. Etain

**Affiliations:** ^1^Université Paris Cité, INSERM UMR-S 1144, Optimisation Thérapeutique en Neuropsychopharmacologie OTeN, Paris; ^2^Fondation FondaMental; ^3^Université Paris Est Créteil, INSERM U955, IMRB, Translational NeuroPsychiatry Laboratory; ^4^AP-HP, Hôpitaux Universitaires Henri Mondor, Département Médico-Universitaire de Psychiatrie et d’Addictologie (DMUIMPACT), Fédération Hospitalo-Universitaire de Médecine de Précision en Psychiatrie (FHU ADAPT), Créteil; ^5^Centre Hospitalier Charles Perrens, Laboratoire NutriNeuro (UMR INRA 1286), Université de Bordeaux, Bordeaux; ^6^Pôle de Psychiatrie, Assistance Publique Hôpitaux de Marseille; ^7^INT-UMR7289, CNRS Aix-Marseille Université, Marseille; ^8^Université Montpellier, Montpellier; ^9^Department of Emergency Psychiatry and Acute Care, CHU Montpellier, IGF, Univ. Montpellier, CNRS, INSERM, Créteil; ^10^Department of Emergency Psychiatry and Acute Care, CHU Montpellier, IGF, Univ. Montpellier, CNRS, INSERM, Montpellier; ^11^AP-HP, Groupe Hospitalo-Universitaire AP-HP Nord, DMU ESPRIT, Service de Psychiatrie et Addictologie, Hôpital Louis Mourier, Colombes; ^12^Université de Paris, Inserm UMR1266, Sorbonne Paris Cité, Faculté de Médecine, Paris; ^13^Université de Lorraine, Centre Psychothérapique de Nancy, Inserm U1254, Nancy; ^14^Pôle de Psychiatrie, Centre Hospitalier Princesse Grace, Monaco; ^15^Centre Hospitalier de Versailles, Service Universitaire de Psychiatrie d’Adulte et d’Addictologie, Le Chesnay; ^16^Equipe DisAP-PsyDev, CESP, Université Versailles Saint- Quentin-en-Yvelines – Paris-Saclay , Inserm, Villejuif; ^17^Université Grenoble Alpes, Inserm, U1216, CHU Grenoble Alpes, Grenoble Institut Neurosciences, Grenoble; ^18^Centre Hospitalier et Universitaire, Département de Psychiatrie, Université Clermont Auvergne, CNRS, Clermont Auvergne INP, Institut Pascal (UMR 6602), Clermont-Ferrand; ^19^Service de Psychiatrie de l’Adultre, CIC-1431 INSERM, CHU de Besançon, Laboratoire de Neurosciences, UFC, UBFC, Besançon; ^20^AP-HP, Groupe Hospitalo-Universitaire AP-HP Nord, DMU Neurosciences, Hôpital Fernand Widal, Département de Psychiatrie et de Médecine Addictologique, Paris, France

## Abstract

**Introduction:**

A 10-15 years decrease in life expectancy has been observed in individuals with bipolar disorder (BD) and has been associated with premature cellular aging, but mechanisms involved remain unclear. Our team recently identified a subgroup of young individuals with prematurely shortened telomere length (TL).

**Objectives:**

The aims of the present study were to replicate this observation in a larger sample and to analyze the expression levels of genes associated with age or TL in a subsample of these individuals.

**Methods:**

TL was measured by qPCR using peripheral blood DNA from 542 individuals with BD. Clustering analyzes were performed with age and TL as classification variables to identify similar groups.

Gene expression of 29 genes, including 20 associated with age and 9 with TL, was analyzed by RT-qPCR using peripheral blood RNA in a subgroup of 129 individuals. Gene expressions were compared between groups obtained from the previous clustering analyzes by Kruskal-Wallis and Mann-Whitney tests.

**Results:**

Clustering analyzes identified 3 subgroups and replicated the clustering previously described: a subgroup of aged individuals with a low TL (mean age : 51.73 years ; mean TL : 2), a subgroup of young individuals with a high TL (mean age : 29.02 years ; mean TL : 4.36) and a subgroup of young individuals but with a low TL (mean age : 29.64 years ; mean TL : 1.96). None of the tested clinical variables were significantly associated with this subgroup.

Furthermore, gene expression level analyzes showed that only *POT1* expression was different between the two subgroups of young individuals, with a downregulation of *POT1* expression in the subgroup with a lower TL level. POT1 is a protein involved in the maintenance of TL. POT1 binds to another protein TPP1 allowing the recruitment of telomerase, the enzyme which extends TL. Our hypothesis is that in the subgroup presenting a lower *POT1* expression, the POT1-TPP1 complex cannot form and thus prevents telomerase recruitment and TL elongation.

**Conclusions:**

This study confirms, on a larger sample, the existence of a subgroup of young individuals with BD presenting accelerated cellular aging. The observed decrease of *POT1* expression level suggests a newly described cellular mechanism in individuals with BD, that may contribute to telomere shortening.

**Disclosure of Interest:**

None Declared

